# Optimisation of a double-centrifugation method for preparation of canine platelet-rich plasma

**DOI:** 10.1186/s12917-017-1123-3

**Published:** 2017-06-26

**Authors:** Hyeok-Soo Shin, Heung-Myong Woo, Byung-Jae Kang

**Affiliations:** College of Veterinary Medicine and Institute of Veterinary Science, Kangwon National University, Chuncheon, 24341 Republic of Korea

**Keywords:** Dog, Double centrifugation, Platelet-derived growth factor-BB, Platelet-rich plasma, Platelet recovery

## Abstract

**Background:**

Platelet-rich plasma (PRP) has been expected for regenerative medicine because of its growth factors. However, there is considerable variability in the recovery and yield of platelets and the concentration of growth factors in PRP preparations. The aim of this study was to identify optimal relative centrifugal force and spin time for the preparation of PRP from canine blood using a double-centrifugation tube method.

**Methods:**

Whole blood samples were collected in citrate blood collection tubes from 12 healthy beagles. For the first centrifugation step, 10 different run conditions were compared to determine which condition produced optimal recovery of platelets. Once the optimal condition was identified, platelet-containing plasma prepared using that condition was subjected to a second centrifugation to pellet platelets. For the second centrifugation, 12 different run conditions were compared to identify the centrifugal force and spin time to produce maximal pellet recovery and concentration increase. Growth factor levels were estimated by using ELISA to measure platelet-derived growth factor-BB (PDGF-BB) concentrations in optimised CaCl_2_-activated platelet fractions.

**Results:**

The highest platelet recovery rate and yield were obtained by first centrifuging whole blood at 1000 g for 5 min and then centrifuging the recovered platelet-enriched plasma at 1500 g for 15 min. This protocol recovered 80% of platelets from whole blood and increased platelet concentration six-fold and produced the highest concentration of PDGF-BB in activated fractions.

**Conclusions:**

We have described an optimised double-centrifugation tube method for the preparation of PRP from canine blood. This optimised method does not require particularly expensive equipment or high technical ability and can readily be carried out in a veterinary clinical setting.

**Electronic supplementary material:**

The online version of this article (doi:10.1186/s12917-017-1123-3) contains supplementary material, which is available to authorized users.

## Background

Investigations of the morphologic and biologic features of platelets have established their important role in wound healing through the release of various growth factors. These factors, which include platelet-derived growth factor (PDGF), transforming growth factor beta, vascular endothelial growth factor, epidermal growth factor and osteoconductive proteins (fibrin, fibronectin and vitronectin), are contained in platelet α granules and are released when platelets are activated or destroyed [1–3]. The release of these growth factors into damaged tissue facilitates tissue regeneration by stimulating cell proliferation, cell migration, angiogenesis and vascular ingrowth [4, 5]. Furthermore, platelets also release antimicrobial, anti-inflammatory and analgesic factors [6–8].

The use of platelet-rich plasma (PRP) to deliver growth factors has emerged as a convenient method for promoting wound healing and tissue regeneration. The autologous nature of PRP preparations makes them safer than other allogenic cell-based regenerative therapies and less expensive. As a result, PRP therapy is seeing widespread use in human medicine, with applications in dentistry, orthopaedic surgery, cardiac surgery, ophthalmology and plastic surgery [9–12]. PRP is also increasingly being used in veterinary medicine [13–15], prompting the need for development of simple and reproducible methods for preparation of high-quality PRP for use in veterinary patients.

Despite the popularity and advantages of PRP, there are conflicting reports in the literature regarding its efficacy [9–14, 16]. These differences may in part be attributable to variations in the composition of PRP preparations with respect to platelet yield, concentrations of beneficial growth factors and the presence of other blood components, particularly leukocytes [17–19]. Tube methods employing differential centrifugation to separate and concentrate platelets are most convenient clinical settings because they are low cost and do not require specific technical expertise. Considerable research effort has been focused optimising PRP protocols reproducible recovery, yield and cellular composition. Most of these studies have focused on PRP prepared from human blood [20–22]; relatively fewer studies have addressed optimisation of PRP for use in veterinary medicine [23, 24]. Several commercial products are available for producing PRP from human blood, but they have been found to produce variable results when used to prepare PRP from canine blood [25].

The purpose of this study was to identify optimal centrifugal force and spin times for the preparation of PRP from canine whole blood using a double-centrifugation tube method. The goal was to identify run conditions that will reproducibly recover large numbers of platelets capable of releasing large amounts of growth factor when activated and that are relatively free of other contaminating blood components. To determine which run conditions were optimal, preparations were assessed for platelet recovery and yield, concentrations of white blood cells (WBC) and red blood cells (RBC) and the amount of PDGF-BB released from activated platelets.

## Methods

### Experimental animals

Twelve, clinically healthy adult male beagles, bred for research purposes (Dreambio, Seoul, Republic of Korea), were included in this study. Dogs were evaluated at the Kangwon National University Veterinary Teaching Hospital by means of physical examination, thoracic and abdominal radiography, complete blood count (CBC), serum biochemistry profile and blood smear microscopic analysis by a veterinarian and were deemed healthy. All dogs in this experiment had normal blood count and morphological features. They ranged from 14.3–17.8 months of age. Body weights ranged were 9.3–10.1 kg. The dogs were individually housed in stainless steel cages at conventional housing facilities with controlled temperature (21 °C) and humidity of 55%. They received standard care and water and food. All dogs were under the care of a licensed veterinarian.

### Blood collection

Whole blood was collected from the jugular vein of each dog using a 21-gauge multiple-use needle into blood collection tubes containing 1.5 mL anticoagulant acid citrate dextrose-A solution (BD Vacutainer system, Plymouth, UK). No more than 90 mL of blood was drawn from each dog per collection, with at least 4 weeks between collections.

Sampled whole blood was divided into 9-mL aliquots that were centrifuged in 15 mL conical centrifuge tubes. Centrifugation and analysis of samples was initiated within 1 h of blood collection.

### Preparation of platelet concentrates

A double-spin preparation method was used for all samples. Samples were centrifuged at room temperature in a Hanil MF-550 centrifuge (HanilScience, Kimpo, Korea) and centrifugal brake was not applied. For the first spin, samples were centrifuged at one of 5 different RCFs (100, 200, 300, 500 or 1000 g) for 5 and 10 min at room temperature (RT), for a total of 10 RCF-time comparisons, with four replications for each comparison. Four replications were conducted on different days using blood drawn from different individuals, and CBC of both whole blood and PRP1 used in each experiment were performed.

After the first centrifugation, the whole plasma fraction above the buffy coat (PRP1) was transferred to an empty tube, its volume was measured, and after mixing this fraction well, a 1-mL aliquot was taken for CBC.

The second centrifugation step was performed with PRP1 prepared using the first-centrifugation RCF-time condition that recovered the highest percentage of platelets. The PRP1 fraction was divided into 1-mL aliquots, which were centrifuged at RT at either 500, 1000, 1500 or 3000 g for 10, 15 or 20 min, for a total of 12 RCF-time comparisons.

The second centrifugation produced a platelet-poor plasma (PPP) supernatant and a platelet-enriched pellet. A volume of 700 μL of PPP supernatant was removed, and the pellet was resuspended in 300 μL PPP; this concentrated platelet fraction was designated as PRP2.

### Platelet activation and PDGF-BB quantification

There are so many activated PDGF such as EGF, PDGF and TGF-β. Because PDGF-BB is not included in the plasma, its concentration correlates well with the quantity of activated platelets. Therefore, PDGF-BB was chosen as an index of platelet activation. PDGF-BB levels were assessed in activated PRP1 and PRP2 samples as well as plasma that had been prepared by centrifuging whole blood at 1000 g for 5 min. To activate samples, they were activated using 20 mM CaCl_2_ and incubated at 37 °C for 1 h followed by 4 °C for 16 h. Samples were then centrifuged at 3000 g for 20 min at 18 °C, and the supernatant collected for PDGF-BB measurement.

The supernatant for PDGF-BB measurement was diluted 20-fold with diluent buffer in kit and quantified by enzyme-linked immunosorbent assay (ELISA) using antibodies against canine PDGF-BB (Raybiotech, Norcross, GA, USA) according to the manufacturer’s instructions. The detection limits ranged from 4 pg/mL to 1200 pg/mL, the interassay coefficient was <12%, and the intraassay coefficient was <10%.

### Haematological analysis

A CBC of whole blood and PRP1 and PRP2 fractions was carried out using an automated laser flow cytometry haematology analyser (ProCyte Dx, IDEXX, ME, USA).

### Statistical analysis

Statistical analysis was performed using Prism 5.00 software (Graphpad Software Inc. San Diego, CA, USA). Results are presented as mean ± standard deviation. Statistical significance was assessed using the Kruskal–Wallis test followed by the Mann–Whitney *post-hoc* test (to compare all pairs in group). *P* < .05 was considered significant for all tests.

## Results

The 12 dogs from which blood was collected weighed between 9.3 and 10.1 kg. No significant weight changes and side effects occurred for any dog during the course of the study.

### PRP1 after first centrifugation step

Table 1 shows the results for the 10 different RCF-time run conditions used for the first centrifugation. The percent of platelets recovered from whole blood progressively increased as the RCF increased when centrifugation time was held constant. Platelet recovery also increased when RCF was held constant but centrifugation time increased 5 min to 10 min for every RCF except the 1000 g condition. The greatest recovery was achieved with run condition 9 (1000 g, 5 min). Comparison of mean percent recoveries of run conditions 1–5 and 7 were all significantly lower than the recovery of condition 9. The mean percent platelet recoveries for conditions 6, 8, 9 and 10 were not significantly different. Furthermore, only 8.18 ± 0.98% of the whole blood WBCs remained in PRP1 obtained through condition 9, which was not significantly different from the WBC recovery rate of PRP1 obtained under conditions 1–8, and it was significantly lower than that of condition 10. Therefore, because the centrifugation time was shorter, condition 9 was chosen as optimal for the first centrifugation step. This condition was used to prepare PRP1 samples for use in optimising the second centrifugation step. (See Additional file 1)

**Table 1:** Platelet recovery after total blood centrifugation

Run	Parameters		Recovery (%)	*P* value
	RCF (× g)	Centrifugation time (minutes)		
1	100	5	16.33 ± 10.92	0.0286^*^
2	100	10	38.53 ± 15.46	0.0286^*^
3	200	5	31.05 ± 14.18	0.0286^*^
4	200	10	55.51 ± 12.14	0.0286^*^
5	300	5	42.62 ± 10.22	0.0286^*^
6	300	10	73.00 ± 12.10	0.1143
7	500	5	72.69 ± 6.90	0.0286^*^
8	500	10	76.62 ± 8.57	0.1143
9	1000	5	92.28 ± 4.88	-
10	1000	10	82.77 ± 8.35	0.3429

Values expressed as mean ± standard deviation; RCF, relative centrifugal force. Asterisk (*) indicates a recovery significantly different (*P* < .05) from condition 9, which produced the highest recovery

### PRP2 after second centrifugation step

The purpose of second centrifugation step was to further concentrate platelets by reducing the volume of PPP. To identify the optimal RCF-time run conditions for the second centrifugation, PRP1 prepared by condition 9 (1000 g, 5 min) was used. The amount of PRP1 obtained by the first centrifugation varied depending on the packed cell volume of each individual, and mean volume of PRP1 was 4.2 ± 0.32 mL. The PRP1 fraction was divided into 1-mL aliquots and samples were subjected to 12 different centrifugation conditions to obtain PRP2 with four replications for each comparison.

Table 2 shows the percent recovery of platelets relative to PRP1 and yield (fold increase) relative to whole blood at each run condition. Lowest RCF resulted in less percent recovery. Recovery increased with increasing RCF and duration of centrifugation. Recovery was substantially reduced following centrifugation at 3000 g for 20 min due to platelet aggregation. Condition 8 (1500 g, 15 min) resulted in both highest recovery and increase in platelet concentration. Statistical analysis revealed that the recovery and yield for condition 8 was not significantly different from results obtained using condition 5 (1000 g, 15 min), condition 9 (1500 g, 20 min) or condition 10 (3000 g, 10 min). Based on these results, conditions 5, 8, 9 and 10 were chosen for additional assessment and each of these 4 run conditions were replicated 8 times.

**Table 2:** Platelet yield and recovery after the second centrifugation step

Run	Parameters			*P* value	Recovery (%)	*P* value
	RCF (× g)	Centrifugation time (min)	Yield (fold)			
1	500	10	4.42 ± 0.84	0.0286^*^	63.21 ± 4.66	0.0286^*^
2	500	15	4.65 ± 0.76	0.0286^*^	66.65 ± 3.64	0.0286^*^
3	500	20	5.60 ± 0.49	0.1143	74.50 ± 1.51	0.0286^*^
4	1000	10	5.41 ± 0.37	0.0286^*^	72.06 ± 2.68	0.0286^*^
5	1000	15	5.94 ± 0.36	0.3429	82.23 ± 6.51	0.4897
6	1000	20	5.65 ± 0.13	0.0286^*^	78.08 ± 2.85	0.0286^*^
7	1500	10	5.80 ± 0.48	0.1143	81.96 ± 2.58	0.0286^*^
8	1500	15	6.48 ± 0.46	-	91.70 ± 4.49	-
9	1500	20	5.93 ± 0.28	0.3429	86.73 ± 1.54	0.4897
10	3000	10	5.82 ± 0.39	0.3429	85.06 ± 3.28	0.1143
11	3000	15	5.75 ± 0.49	0.3429	85.87 ± 1.65	0.0286^*^
12	3000	20	4.34 ± 1.06	0.1143	64.79 ± 13.73	0.0286^*^

Values expressed as mean ± standard deviation; RCF, relative centrifugal force. Asterisk (*) indicates a recovery or yield significantly different (*P* < .05) from condition 8, which produced the highest recovery and yield

**Fig. 1:**
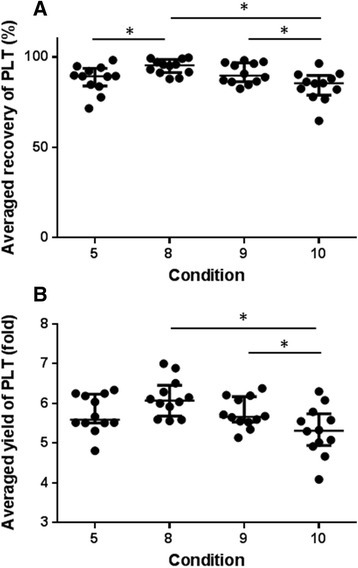
Scatter plots for platelet recovery and yield of selected 4 conditions after the second centrifugation step. **a** Percent platelet recovery relative to PRP1 and (**b**) yield expressed as fold increase relative to whole blood. Data are presented by groups, the line represents the median of the group; error bars represent the interquartile range. Asterisk (*) indicates a recovery or yield significantly different (*P* < .05) between the two groups. Condition 5, 1000 g, 15 min; condition 8, 1500 g. 15 min; condition 9, 1500 g, 20 min; condition 10, 3000 g, 10 min

Mean percent platelet recoveries relative to PRP1 and yields relative to whole blood under run conditions 5, 8, 9 and 10 are shown in Fig. 1a and b, respectively. Platelet recovery and yield were once again the highest when samples were centrifuged at 1500 g, 15 min (condition 8). The platelet recovery rate for condition 8 was significantly higher than those for conditions 5 and 10 but not higher than that for condition 9. The platelet yield for condition 8 was significantly greater than that for condition 10, but it was not significantly greater than the yield for conditions 5 and 9 (Fig. 1b). There was no statistically significant difference between conditions 8 and 9, but condition 8 could shorten centrifugation time by 5 min. Based on these results, condition 8 (1500 g, 15 min) was chosen as optimal for the second centrifugation step.

Table 3 shows the concentrations of platelets, RBC, total WBC and lymphocytes in whole blood and in PRP2 prepared using the centrifugation conditions identified as optimal for platelet recovery and yield (i.e. initial centrifugation of whole blood at 1000 g for 5 min to obtain PRP1, followed by centrifugation of PRP1 at 1500 g for 15 min). This centrifugation protocol recovered approximately 80% of the platelets from whole blood and increased platelet concentration approximately 6-fold. The concentration of platelets in the PRP preparation was 1748 ± 193 × 10^3^ cells/μL, which was 6.1 ± 0.4 times higher than the concentration in the original whole blood sample. The PRP preparations contained negligible concentrations of RBCs, and WBC contents were 8.5 ± 1% neutrophils, 15.5 ± 4% monocytes and 73 ± 7% lymphocyte. (See Additional file 1)

**Table 3:** Descriptive results after double centrifugation

Variables	Whole blood	PRP1	PRP2
Red blood cells (cells × 10^6^/μL)	5.8 ± 0.23	0.03 ± 0.05*	0.065 ± 0.02^#^
Lymphocytes (cells × 10^3^/μL)	2.3 ± 0.54	1.05 ± 0.77	3.71 ± 2.4
Neutrophil (cells × 10^3^/μL)	7.11 ± 1.67	0.15 ± 0.17*	0.44 ± 0.39^#^
Monocyte (cells × 10^3^/μL)	0.7 ± 0.15	0.2 ± 0.13	0.79 ± 0.57
White blood cells (cells × 10^3^/μL)	10.32 ± 2.4	1.45 ± 0.87*	5.09 ± 3.39^#^
Platelets (cells × 10^6^/μL)	289 ± 35	523 ± 56*	1748 ± 193^#^

Values expressed as mean ± standard deviation (*n* = 12). Asterisk (*) indicates a significant difference (*P* < .05) between platelet-rich plasma-1 and whole blood. # indicates a significant difference (*P* < .05) between platelet-rich plasma-2 and whole blood

### PDGF-BB quantification

**Fig. 2:**
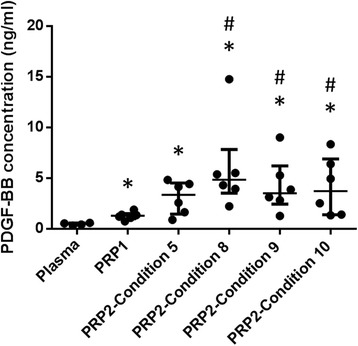
Scatter plots for PDGF-BB concentrations in plasma, PRP1 and PRP2 * Indicates a statistically significant difference (*P* < 0.05) compared with plasma. # Indicates a statistically significant difference (*P* < 0.05) compared with PRP1. Data are presented by groups, the line represents the median of the group; error bars represent the interquartile range. Condition 5, 1000 g, 15 min; condition 8, 1500 g. 15 min; condition 9, 1500 g, 20 min; condition 10, 3000 g, 10 min

To determine the effects of the centrifugation protocol on the level of platelet-associated growth factors in activated fractions, concentrations of a representative growth factor, PDGF-BB, were measured in plasma, PRP1, and in the various PRP2 fractions prepared using conditions 5, 8, 9, 10 and 11. Figure 2 shows that condition 8 (1500 g, 15 min), which produced the highest recovery and yield of platelets, correspondingly also had the highest concentration of PDGF-BB. PRP2 prepared under condition 8 was the only PRP2 fraction that had a significantly higher PDGF-BB concentration than plasma or PRP1.

## Discussion

In this study, we prepared PRP from canine blood using a double-centrifuge tube method. We found that the following protocol produced the greatest recovery and yield of platelets and highest concentrations of PDFG-BB in PRP preparations:

1. Collect blood samples in 10-mL tubes containing 3.2% sodium citrate to prevent coagulation. Retain a 0.5-mL small aliquot for initial CBC.
2. Centrifuge the blood sample at 1000 g for 5 min at RT.
3. Transfer the upper plasma and platelet fraction (PRP1) into a sterile tube and measure the volume. Retain an aliquot for determination of PRP1 platelet concentration.
4. Centrifuge the PRP1 fraction at 1500 g for 15 min at RT.
5. Draw off two-thirds of the PPP supernatant and resuspend the platelet-containing pellet PRP2 in the remaining PPP. Measure the PRP2 volume and take an aliquot for determination of PRP2 platelet concentration

The purpose of the first spin is to separate as many platelets as possible from the whole blood. Centrifugation separates blood components on the basis of their density gradients. Platelets are the lightest components, followed by WBCs, and RBCs are heaviest [26]. Therefore, RCF and time must be appropriate to obtain optimal separation of blood components. In this study, a low RCF and short duration of centrifugation was insufficient to obtain good separation of platelets from other blood cells, resulting in low recovery. The highest platelet recovery in conditions 9 and 10, which were the highest RCF conditions, indicated that the 1000-g condition was the most suitable condition to sink RBCs and WBCs and separated platelets at the upper layer. However, in condition 10, the WBC recovery rate was also high because the high RCF and long centrifugation time caused the platelet to overlap with the WBC layer.

The purpose of the second spin is to separate platelets from plasma, which is accomplished by pelleting the platelets and discarding most of the PPP supernatant. To maximise platelet recovery and yield, a higher RCF and longer duration of centrifugation than used in the first spin were needed. However, we found that if the RCF time was too high, the recovery and yield were reduced due to platelet aggregation. Aggregated platelets were not resuspended in PPP, thus resulting in substantial platelet count reduction. The blood analyser detects aggregated platelets in PRP2; although the actual numbers of platelets were not decreased, as aggregated (activated) platelets release growth factors during the preparation process, the amount of growth factors in the final product was found to decrease. We concluded that the optimal combination was 1000 g for 5 min for the first spin and 1500 g for 15 min for the second spin.

The RCFs we found to be optimal for preparation of PRP from canine blood were higher than those found for human blood, although the centrifugation times were similar [20, 21]. This may be related to haematologic difference between human and dog blood. Canine mean corpuscular volume and mean corpuscular haemoglobin values are lower than human values [27, 28]. These means dog RBCs are smaller and less dense than human RBCs, so relatively higher centrifugal force is required to separate canine blood components.

The optimised protocol increased platelet concentration was 6- to 7-fold relative to the initial concentration in whole blood (Table 2), and final platelet concentration was 1748 ± 193 × 10^3^ platelets μL^−1^ (Table 3). The American Red Cross defines PRP as 5.5 × 10^10^ platelets/50 mL (1100 × 10^3^ platelets μL^−1^) [29]. Furthermore, accumulated data suggests that the PRP products must have at least 300 × 10^3^ platelets μL^−1^ to be therapeutically effective, which is 4- to 5-fold higher concentration than whole blood [30–34]. These recommendations may not be appropriate to every situation; however, because this protocol produces a high yield of platelets, the concentration can adjusted as necessary by manipulating the ratio of PPP to PRP. Franklin et al. [25] recently conducted a study in which they used five commercially available kits to prepare PRP from canine whole blood. The yield and compositions of the canine PRP prepared using these kits varied considerably. The concentration of platelets in the canine PRP prepared using our optimised protocol was higher than the concentrations achieved with any of the commercial systems. Frye et al. [35] also showed PRP, with platelet and leukocyte concentrations similar to ours through the double-spin method using commercial kits, but its disadvantage was that it was expensive.

Using differential centrifugation to prepare platelet concentrates completely free of other blood cell types is impossible because the specific gravities of the various components slightly overlap [36]. However, our optimised protocol resulted in PRP with negligible number of RBCs compared with other studies [20, 21], and only 8.18 ± 0.98% of total blood WBC counts were contained in PRP1. Also, the WBC concentration in the final product was 2-fold lower than the normal range. In particular, the recovery rate of lymphocytes and monocytes from PRP1 obtained with the first spin was higher than that of neutrophils because the lymphocyte and monocyte weights were relatively light and were located in the upper layer near the platelets after centrifugation.

The role of WBC in PRP therapy remains a matter of disagreement in the literature. Some studies have suggested that increased WBC concentrations in PRP can impede tissue regeneration and healing. The presence of WBC in PRP has been associated with increased scar tissue and collagen degradation ex vivo. High WBC concentration may affect negatively with matrix production in tendons [37–39]. To prevent potential negative effects, it has been suggested that platelet preparations only be transfused if they contain fewer than 5 × 10^5^ WBC/μL [40]. However, some research has suggested that some WBC contamination may be beneficial because WBC can increase growth factor secretion. PRP that contained enough number of WBCs was recommended as another therapeutic purpose because WBC triggered growth factor secretion by the tissue being treated with the PRP [41, 42]. There is not enough data in the literature to determine whether WBCs are beneficial to PRP therapy [29]. Additional studies will be necessary to optimise PRP with regard to their WBC content. Previously, Perazzi et al. [23] performed PRP preparation study using the double-centrifugation method, which is similar to ours. In their study, platelet concentration at least four times the baseline was obtained in 10 out of 12 dogs (83.3% of cases), but WBC concentration was also higher than that at the baseline. Furthermore, they did not measure growth factor concentration in PRP from their protocol; thus, it remained unknown that high platelet number represented high growth factor concentration in PRP.

Among the numerous growth factors present in platelet α granules, PDGF-BB was selected as an index of activated platelets in this study because it is only secreted by activated platelets and not contained in plasma. Thus, its concentration represented the extent to which platelets were activated. PDGF-BB concentration was highest in PRP prepared by the optimised method. Although the PRP that had the highest platelet concentration also had the highest PDGF-BB level, platelet concentration is not the only determinant of growth factor concentration. The type of anticoagulant used during blood collection, the ratio of blood to anticoagulant, the type of activating agent and the activation protocol can all have an influence of the concentration of growth factor in PRP [20]. A previous study showed that platelets released the highest PDGF-BB when they were activated with CaCl_2_ [43]_._ Further, the amount of TGF-β released by platelets activated by thrombin or CaCl_2_ was similar [43]. In addition, the process of obtaining autologous thrombin is complicated, but activation using CaCl_2_ is inexpensive and easy. Therefore, we used CaCl_2_ to activate platelets. However, we did not measure whether platelets were fully activated in the present study. Among the PRPs with the same number of platelets, the amount of growth factors will depend on the number of activated platelets. To activate as much platelets as possible, various agonist (collagen, ADP and thrombin, etc.) and ratio between PRP and agonist should be further studied.

One potential limitation of using centrifugation to prepare PRP is that there is a risk for bacterial contamination during transfers between tubes. However, this risk can be greatly reduced if transfers are conducted in a laminar flow hood under sterile conditions. Despite this potential drawback, the protocol we have described in this study does not require particularly expensive equipment or high technical ability and can readily be carried out in a veterinary clinical setting.

## Conclusions

The PRP therapy, research and associated-industry are burgeoning fields in veterinary medicine. For the effective and reliable clinical application of PRP in veterinary medicine, more studies are needed with regard to PRP characteristics as well as patient factors that influence the effectiveness of PRP. Our protocol meets the guideline of The American Red Cross and recommendations of other articles associated with the number of platelets in PRP. The PRP from our optimised protocol may produce consistently reproducible results and it will help advance comparative research and therapeutic use of PRP-related products in veterinary medicine.

## Additional file

Additional file 1: WBC, neutrophil, lymphocyte and monocyte recovery after total blood centrifugation. The recovery rate of WBC, neutrophil, lymphocyte and monocyte for whole blood of each PRP1 obtained after the first centrifugation under 10 different conditions are shown in the table. (DOCX 18 kb)

## Abbreviations

ACD: Acid citrate dextrose
EGF: Epidermal growth factor
MCH: Mean corpuscular haemoglobin
MCV: Mean corpuscular volume
PDGF-BB: Platelet-derived growth factor-BB
PPP: Platelet-poor plasma
PRP: Platelet-rich plasma
RBC: Red blood cell
RCF: Relative centrifugal force
TGF: Transforming growth factor
VEGF: Vascular endothelial growth factor
WBC: White blood cell
