# Supplementary material for: Optimisation of a double-centrifugation method for preparation of canine platelet-rich plasma
Source: BMC Vet Res. 2017 Jun 26;13:198. doi: 10.1186/s12917-017-1123-3 (PMC5485745; doi:10.1186/s12917-017-1123-3)
Supplement: Additional file 1: — WBC, neutrophil, lymphocyte and monocyte recovery after total blood centrifugation. The recovery rate of WBC, neutrophil, lymphocyte and monocyte for whole blood of each PRP1 obtained after the first centrifugation under 10 different conditions are shown in the table. (DOCX 18 kb) [file 12917_2017_1123_MOESM1_ESM.docx]

| Run | **Parameters** | | **WBC** | | **Neutrophil** | | **Lymphocyte** | | **Monocyte** | |
| --- | --- | --- | --- | --- | --- | --- | --- | --- | --- | --- |
|  | **RCF (x g)** | **Centrifugation time (min)** | **Recovery (%)** | ***P* value** | **Recovery (%)** | ***P* value** | **Recovery (%)** | ***P* value** | **Recovery (%)** | ***P* value** |
| 1 | 100 | 5 | 6.63 ± 4.17 | 0.4857 | 3.33 ± 3.04 | 0.3429 | 14.18 ± 6.73 | 0.1143 | 14.65 ± 6.06 | 0.6571 |
| 2 | 100 | 10 | - 1. ± 9.04 | >0.9999 | 5.48 ± 7.66 | 0.8286 | 24.43 ± 14.60 | 0.6571 | 25.89 ± 14.12 | 0.6571 |
| 3 | 200 | 5 | 6.25 ± 1.68 | 0.3429 | 1.85 ± 1.22 | 0.4857 | 18.50 ± 4.75 | 0.1143 | 12.92 ± 2.52 | 0.1143 |
| 4 | 200 | 10 | 8.51 ± 0.94 | 0.8286 | 1.05 ± 0.32 | 0.8286 | 28.55 ± 2.47 | 0.8286 | 20.06 ± 3.08 | 0.6571 |
| 5 | 300 | 5 | 9.37 ± 3 | 0.3429 | 2.13 ± 2.14 | >0.9999 | 28.86 ± 7.31 | 0.6571 | 22.43 ± 6.56 | 0.8286 |
| 6 | 300 | 10 | 8.34 ± 3.05 | >0.9999 | 1.57 ± 1.39 | 0.8286 | 26.14 ± 6.83 | >0.9999 | 14.24 ± 2.42 | 0.6571 |
| 7 | 500 | 5 | 6.94 ± 1.53 | 0.4857 | 1.65 ± 0.9 | 0.4857 | 21.40 ± 4.21 | 0.6571 | 8.98 ± 2.74 | 0.0571 |
| 8 | 500 | 10 | 7.67 ± 1.34 | 0.6571 | 1.87 ± 0.33 | 0.1143 | 22.69 ± 3.85 | 0.6571 | 10.02 ± 1.39 | 0.1143 |
| 9 | 1000 | 5 | 8.18 ± 0.98 | - | 1.17 ± 0.52 | - | 27.98 ± 10.41 | - | 21.83 ± 10.76 | - |
| 10 | 1000 | 10 | 34.49 ± 9.85 | 0.0286* | 26.39 ± 9.95 | 0.0286* | 60.58 ± 18.23 | 0.0571 | 51.14 ± 31.42 | 0.4857 |

**WBC, neutrophil, lymphocyte and monocyte recovery after total blood centrifugation**

Values expressed as mean ± standard deviation; RCF, relative centrifugal force. Asterisk (*) indicates a recovery significantly different (*P* < .05) from condition 9, which produced the highest recovery.
